# Apixaban-Induced Hepatotoxicity

**DOI:** 10.7759/cureus.23879

**Published:** 2022-04-06

**Authors:** Umair Ansari, Zahra Asghar, Meghan Oswald, Hong Ng

**Affiliations:** 1 Internal Medicine, Mather Hospital, Northwell Health, Port Jefferson, USA; 2 Psychiatry, Mather Hospital, Northwell Health, Port Jefferson, USA

**Keywords:** hepatotoxicity, general pharmacology, adult gastroenterology, lft - liver function tests, apixaban, general internal medicine, cardiology research

## Abstract

Apixaban is widely used to prevent and manage thromboembolic disease. Due to it being fairly new in the market, we are still understanding its complete risk profile. We present a case of a 61-year-old female with no prior history of liver disease, who developed severe transaminitis shortly after the initiation of apixaban and started trending down after its discontinuation.

## Introduction

Apixaban is a direct oral anticoagulant (DOAC) that works by inhibiting factor Xa [1]. It is widely used to prevent and manage thromboembolic disease; it was approved for non-valvular atrial fibrillation in 2012 and venous thromboembolism prophylaxis post knee and hip replacement in 2014 [2]. Although less so than vitamin K antagonists, the most common side effect is bleeding [3]. Despite being metabolized in the liver, apixaban has rarely been shown to cause hepatotoxicity. We present a rare case of apixaban-induced hepatotoxicity in a patient with no history of liver disease.

## Case presentation

Our patient is a 61-year-old female with a past medical history of hypertension and Sjögren's syndrome who presented to the hospital with the chief complaint of anhedonia. She was admitted to the inpatient psychiatry unit for the working diagnosis of major depressive disorder. On admission, her labs and vitals were were normal, with aspartate aminotransferase (AST) of 21 U/L, alanine aminotransferase (ALT) of 25, alkaline phosphatase (ALP) of 93 U/L, and total bilirubin was 0.9 mg/dL. Her acetaminophen and alcohol levels were <5 ug/mL and <10 mg/dL, respectively. Her home medications included sertraline 50 mg daily, gabapentin 300 mg daily, bupropion 300 mg/24 hours extended-release daily, fosinopril 40 mg daily, and clonazepam 0.5 mg two times a day as needed. She had not been started on prophylaxis for venous thromboembolism on admission, as she was ambulating. During the first seven days of admission, her home medications were titrated down and subsequently discontinued, except the fosinopril. Her sertraline was switched to escitalopram 10 mg daily. 

On day five, her hospital course had been complicated by right leg pain and a lower extremity Doppler showed a right popliteal deep vein thrombosis extending into the calf veins. She was subsequently started on a loading dose of apixaban 10 mg every 12 hours. She was also given tramadol 50 mg as needed for leg pain, which she took intermittently. The internal medicine team was consulted on day 10 for transaminitis found on routine lab work (Table 1). Her vital signs were within normal limits. The patient was asymptomatic. She had no prior history of liver disease or alcohol use. The abdominal exam was unremarkable. An abdominal ultrasound revealed cholelithiasis, gallbladder sludge, and a prominent CBD with a diameter of 6.6 mm. Hepatitis panel and further hepatic workup (including iron profile) are illustrated in Table 2 and Table 3, respectively. 

**Table 1:** Vital signs and lab values on day eight

Vital signs		
Parameter	Value	Reference range
Temperature (°F)	97.6	97-99
Blood pressure (bpm)	121/75	<120/80
Heart rate	91	60-100
Respiratory rate (breaths/minute)	17	12-16
Oxygen saturation (SpO2) (%)	N/A	>92
Complete blood count		
Haemoglobin (Hb) (mg/dL)	14.3	13.2-16.6
Hematocrit (Hct) (9%)	45.5	38.3-48.6
White cell count (WBC) (K/ul )	8.86	4.5-11
Coagulation profile		
INR (international normalized ratio)	1.25	0.91-1.12
Prothrombin time (s)	14.3	10.4-12.8
Comprehensive metabolic panel		
Sodium (mmol/L)	140	136-144
Potassium (mmol/L)	3.9	3.6-5.1
Chloride (mmol/L)	101	97-110
Bicarbonate (mmol/L)	29	22-32
Creatinine (mg/dL)	0.73	0.7-1.2
Blood urea nitrogen (mg/dL)	10	8-20
Aspartate aminotransferase (AST) (U/L)	164	0-33
Alanine aminotransferase (ALT) (U/L)	295	0-33
Alkaline phosphatase (ALP) (U/L)	221	35-104
Total bilirubin (mg/dL)	0.6	0-1.2
Gamma glutamyl transferase (GGT) (U/L)	600	5-36

**Table 2:** Hepatitis panel

Lab value	Result
Hepatitis B surface antibody	Non-reactive
Hepatitis B core antibody	Non-reactive
Hepatitis B Surface Ag	Non-reactive
Hepatitis A Antibody	Non-reactive
Hepatitis C antigen	Non-reactive

**Table 3:** Further hepatic workup and iron profile

Lab value	Value	Reference
Cytomegalovirus (mg/dL)	<30.0	<30.0
Antimitochondrial antibody (mg/dL)	<1:20	<1:20
Anti-smooth muscle antibody (mg/dL)	<1:20	<1:20
Alpha-1 antitrypsin antibody (mg/dL)	198	90-200
Ceruloplasmin in serum (mg/dL)	33	16-45
Iron profile		
Ferritin (ng/mL)	963	13-150
Serum Iron (ug/dL)	87	37-145
Total Iron binding capacity (ug/dL)	259.56	250-450
Iron saturation (%)	34	15-50

**Figure 1:**
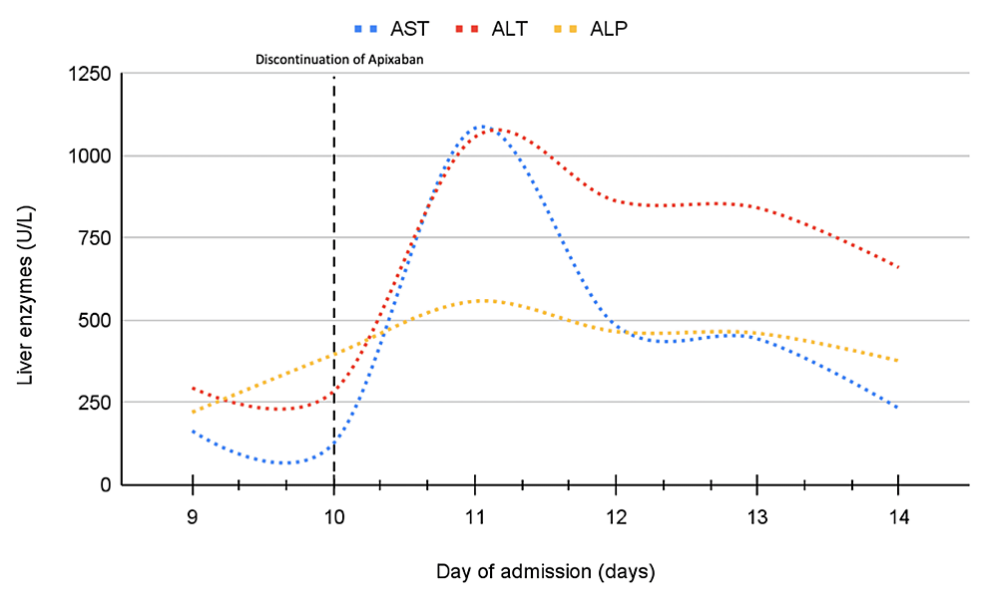
Liver enzymes AST: Aspartate aminotransferase; ALT: Alanine aminotransferase; ALP: Alkaline phosphatase.

The patient’s medications were reviewed. Apixaban, fosinopril, and escitalopram were all discontinued. The liver enzymes peaked on day 11: AST of 1084 U/L, ALT of 1057, ALP of 558 U/L and total bilirubin was 1.5 mg/dL with a direct bilirubin of 0.6 mg/dL, then it started trending down 24 hours after the discontinuation of apixaban (Figure 1). On day 20 of admission, the liver enzymes were as follows: AST of 37 U/L, ALT of 157, ALP of 209 U/L, and total bilirubin was 0.7 mg/dL. Fosinopril was restarted later in the hospital course, without an elevation in liver enzymes. To note, she refused a magnetic resonance cholangiopancreatography (MRCP), to further investigate the abdominal ultrasound findings. 

## Discussion

Our case report illustrates a potentially dangerous side effect of apixaban, not well known in contemporary literature. To our knowledge, there are only a handful of other case reports and observational studies detailing this side effect. In an observational study by Spiller et al. [4], two patients were found to have hepatic transaminase levels greater than 1,000 U/L while on rivaroxaban. Another case report by Clarke et al. [5] demonstrated mild transaminitis with ALT peaking at 199 U/L, AST peaking at 110 U/L, ALP remaining around 72 U/L, and total bilirubin of 2.5 mg/dL with direct bilirubin 1.0 mg/dL in a patient on apixaban. Our case report is unique as the patient was on apixaban and the elevations in liver enzymes were much higher than previous case reports, with AST peaking at 1084 U/L, ALT at 1057 U/L, and ALP at 558 U/L.

Ximelagatra was one of the first DOACs introduced into the market for preventing deep vein thrombosis post knee arthroplasty but was discontinued shortly after they found hepatotoxicity in patients with long exposure during post-market surveillance [6]. Although idiosyncratic hepatotoxicity is a potential concern with any drug; DOACs have been closely monitored for hepatocellular injury ever since. Douros et al. [7] demonstrated that the hazard ratio for serious liver injury in users of DOACs compared with users of vitamin K antagonists was 0.99 (95% confidence interval: 0.68 to 1.45); in patients with prior liver disease, the hazard ratio was 0.68 (95% confidence interval: 0.33 to 1.37).

Reviewing the case, although the exact mechanism of injury remains uncertain, it is likely our patient had an idiosyncratic drug reaction. She had no previous history of liver injury; the transaminitis occurred three days after the initiation of the medication and promptly down trended within 24 hours of discontinuation of apixaban, which is consistent with its half-life of 12 hours [8]. The other two concomitantly discontinued medications namely the fosinopril and escitalopram are unlikely to be the culprit, as the fosinopril was the patient’s home medication and her presenting liver enzymes were within normal limits before apixaban was started. On lab work, the patient's ferritin levels were elevated but serum iron was normal, making a diagnosis of hemochromatosis less likely. Although minimal data exists on antidepressant-induced liver injury, 0.5%-3% of patients treated with antidepressants may develop asymptomatic mild elevation of serum aminotransferase levels [9]. However, escitalopram which has been FDA approved since 2002 is on the list of antidepressants least likely to cause hepatotoxicity. This combination with escitalopram, having a half-life of 27-32 hours, compared to 8-12 hours for apixaban and immediate decrease in liver function with discontinuation of medications points to apixaban as the likely cause of transaminitis. Fosinopril was also restarted later on in the hospital course, without causing further elevations in liver enzymes.

## Conclusions

Due to DOACs being fairly new on the market, we have yet to fully understand their risk profile. Although hepatotoxicity is rare, it is a potentially dangerous yet under-reported side effect of apixaban. Further studies are necessary to further explore this interaction.
